# Cas9 has no exonuclease activity resulting in staggered cleavage with overhangs and predictable di- and tri-nucleotide CRISPR insertions without template donor

**DOI:** 10.1038/s41421-019-0120-z

**Published:** 2019-10-01

**Authors:** Xin Shi, Jia Shou, Mohammadreza M. Mehryar, Jingwei Li, Leyang Wang, Mo Zhang, Haiyan Huang, Xiaofang Sun, Qiang Wu

**Affiliations:** 1 Center for Comparative Biomedicine, MOE Key Lab of Systems Biomedicine, State Key Lab of Oncogenes and Related Genes, Shanghai Cancer Institute, Joint International Research Laboratory of Metabolic & Developmental Sciences, Institute of Systems Biomedicine, Xin Hua Hospital, Shanghai Jiao Tong University, Shanghai, China; 2 The Third Affiliated Hospital of Guangzhou Medical University, Guangzhou, China

**Keywords:** DNA recombination, Bioinformatics

Dear Editor,

Genome editing has found applications in almost every field in biology and medicine and has permeated many aspects of our society. In particular, the clustered regularly interspaced short palindromic repeats (CRISPR)—CRISPR-associated nuclease 9 (Cas9) system—has been widely used in genome editing because of its convenience, simplicity, efficiency, and low cost. It is generally thought that Cas9 cleavage generates blunt-ended double-strand breaks (DSBs) at the 3 base pairs upstream (−3 bp) of protospacer adjacent motif (PAM). In addition, the editing outcomes resulted from several competing cellular DSB repair pathways such as canonical or classic non-homologous end joining (cNHEJ) and microhomology-mediated end joining (MMEJ) are random, imprecise, and unpredictable. Only homologous recombination with a donor template (HDR, homology directed repair) is thought to be precise. However, recent studies revealed that the CRISPR-Cas9 genome editing system is precise and predictable even without donor templates for natural or synthetic targeting sites^1–6^. Specifically, it is firmly established that Cas9-mediated 1-bp insertions are predictable^1–3^^,7–9^ because they are resulted from ligation and polymerase-fill-in of DSB ends with 1-bp overhang from the staggered Cas9 cleavage at the −4 bp position of the noncomplementary strand upstream of the NGG PAM site^1^. However, whether Cas9 mediates 2-bp or 3-bp insertions in a template-free manner and whether they are predictable remain obscure.

Here we performed systematic CRISPR DNA-fragment editing experiments and analyzed deep-sequenced junctional amplicons of chromosomal rearrangements by *Sp*Cas9 programmed with dual sgRNAs for 2-bp or 3-bp insertions (Fig. 1; Supplementary Fig. S1). CRISPR DNA-fragment editing by Cas9 programmed with dual sgRNAs could result in various chromosomal rearrangements, including DNA-fragment deletions, inversions, duplications or circularizations, insertions, and translocations (Supplementary Fig. S1)^10^. Because the rearranged junctional sequences no longer match either of the two sgRNAs, this DNA-fragment editing has the advantage to avoid re-cutting by Cas9 with single sgRNAs^1^. We analyzed 571 amplicon libraries from chromosomal rearrangements resulted from DNA-fragment editing in HEK293T cells by Cas9 with dual sgRNAs (Supplementary Fig. S1). The sgRNAs that we selected include all four combinatorial configurations of the two PAM orientations (NGG-CCN, NGG-NGG, CCN-CCN, and CCN-NGG). Similar to predictable 1-bp insertions^1–3,9^, we found that, among all sixteen possible dinucleotide insertions, there is a strong bias of 2-bp insertions toward the dinucleotide of the −5 and −4 positions on the noncomplementary strand upstream of the NGG PAM site in vivo (Fig. 1). Namely, the inserted dinucleotides tend to be the same as the −5 and −4 positions of the noncomplementary strand upstream of the NGG PAM site. In addition, sequence context influences 2-bp insertions and there tends to favor 2-bp insertions when the −5 and −4 positions are “CA” (Fig. 1; Supplementary Fig. S2). This suggests that donor-template-free 2-bp insertions by the CRISPR-Cas9 system are predictable and that Cas9 endonucleolytically cleaves the noncomplementary strand at −5 position and generates staggered DSB ends with 2-bp overhangs.

**Fig. 1:**
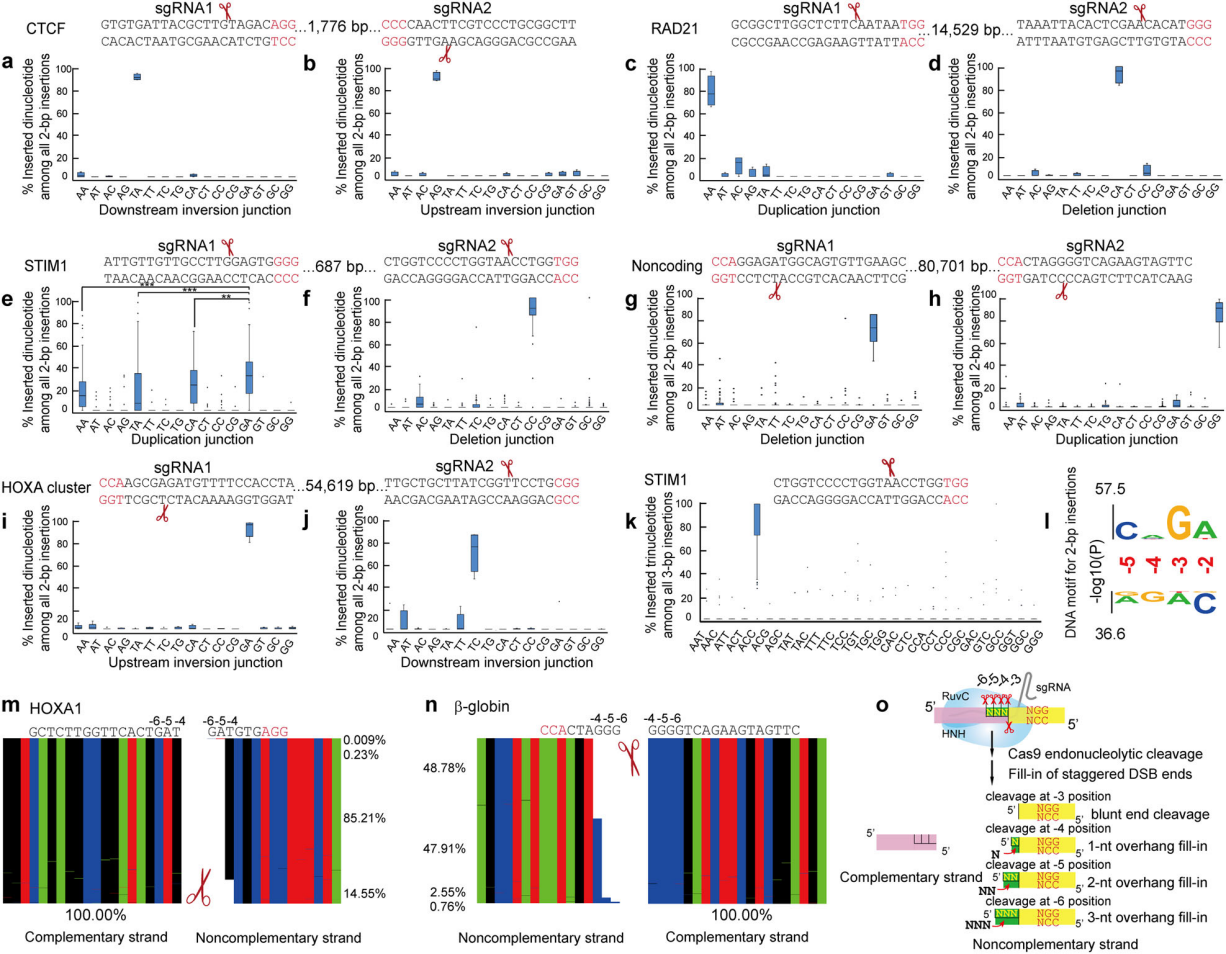
The 2-bp and 3-bp insertions are predictable. **a**–**j** Shown are boxplots of normalized 2-bp insertion frequencies of different targeting sites whose sequences are indicated above each panel by DNA-fragment editing with dual sgRNAs in vivo. For each DNA fragment, chromosomal-rearrangement junctions edited by Cas9 with dual sgRNAs (sgRNA1 and sgRNA2) were amplified by PCR and the amplicons were subjected to NGS. The four PAM configurations with dual sgRNAs (NGG-CCN, NGG-NGG, CCN-CCN, and CCN-NGG) and the size of the edited DNA fragments are also shown. **k** 3-bp insertion frequencies at the STIM1 locus. The boxplot shows the upper quartile, the median, and the lower quartile. Whiskers show the 1.5 times of IQR (Interquartile Range). **l** DNA motif for 2-bp insertion frequencies. Shown are base positions with Bonferroni-corrected *P* values ( < 0.01). **m**, **n** NGS raw sequencing reads showing staggered Cas9 in vitro cleavage patterns of the targeting sites of *HOXA1* (**m**) and *β-globin* (**n**). **o** A fill-in model for predictable di- or tri-nucleotide insertions by the mechanism of staggered Cas9 cleavage. The targeting sequences for all sites are shown with PAM highlighted in red. CTCF CCCTC binding factor, DSB double-strand break, NGS next generation sequencing, sgRNA single guide RNA, PAM protospacer adjacent motif

We first designed two sgRNAs in the NGG-CCN PAM configuration to target a DNA fragment of 1776 bp in the housekeeping gene *CTCF* on the human chromosome 16 (Fig. 1a, b) and analyzed 2-bp insertions at the rearranged chromosomal junctions. Remarkably, we found that almost all of the 2-bp insertions at the downstream junction of DNA-fragment inversion are “TA”. This “TA” insertion is complementary to the dinucleotide at the −4 and −5 positions of the noncomplementary strand upstream of the NGG PAM site for sgRNA1 (Fig. 1a). For the sgRNA2, whose PAM site is in the CCN configuration, we analyzed 2-bp insertions at the upstream junction of DNA-fragment inversion. We found that almost all of the 2-bp insertions are “AG”, which is identical to the dinucleotide at the −5 and −4 positions upstream of the PAM site for sgRNA2 (Fig. 1b).

Next, we analyzed 2-bp insertions at the rearranged junctions of DNA-fragment editing programmed with dual sgRNAs in the NGG-NGG PAM configuration for another housekeeping gene *RAD21*, encoding a subunit of the cohesin complex, in the human chromosome 8. For sgRNA1, we analyzed 2-bp insertions at the junctions of DNA-fragment duplication or circularization and found that almost all of the 2-bp insertions are the same as the dinucleotide “AA” of the −5 and −4 positions upstream of the NGG PAM site (Fig. 1c). Similarly, almost all of the 2-bp insertions at the junctions of DNA-fragment deletion are the same as the dinucleotide “CA” of the −5 and −4 positions upstream of the NGG PAM for sgRNA2 (Fig. 1d). We then analyzed the 2-bp end-joining patterns at the rearranged junctions for the *STIM1* (stromal interaction molecule 1) gene in the human chromosome 11 with another pair of sgRNAs in the NGG-NGG configuration. Although there are no single predominant 2-bp insertions at the junctions of DNA-fragment duplication or circularization, the frequency of “GA” insertions, which corresponds to the −5 and −4 positions for sgRNA1, is still significantly higher than those of the other three prominent 2-bp insertions of “AA”, “TA”, and “CA” (Fig. 1e). Similar to *RAD21*, there are predominant insertions of the dinucleotide “CC” at the junction of DNA-fragment deletions, which is the same as the dinucleotide at the −5 and −4 upstream of the NGG PAM site of the sgRNA2 (Fig. 1f).

We then analyzed 2-bp insertions for editing an 80,701-bp DNA fragment in a noncoding region by Cas9 with dual sgRNAs in the CCN-CCN PAM configuration (Fig. 1g, h). We found that the predominant 2-bp insertion of “GA” at the junctions of DNA-fragment deletion is complementary to the −4 and −5 positions upstream of the PAM site for sgRNA1 (Fig. 1g). Similarly, the predominant 2-bp insertion of “GG” at the junctions of DNA-fragment duplication or circularization is complementary to the −4 and −5 positions upstream of the PAM site for sgRNA2 (Fig. 1h).

We also analyzed 2-bp insertions for the human *HOXA* gene cluster by Cas9 with dual sgRNAs in the CCN-NGG PAM configuration (Fig. 1i, j). We found that the predominant 2-bp insertion of “GA” at the upstream junctions of DNA-fragment inversion is complementary to the −4 and −5 positions upstream of the PAM site for sgRNA1 (Fig. 1i). In addition, the predominant 2-bp insertion of “TC” at the downstream junctions of DNA-fragment inversion is the same as the dinucleotide at the −5 and −4 positions upstream of the NGG PAM site for sgRNA2 (Fig. 1j).

Collectively, we found that 2-bp insertions at junctions of DNA-fragment editing with dual sgRNAs of four possible combinatorial PAM configurations all match the dinucleotide at the −5 and −4 positions of the noncomplementary strand upstream of the PAM site in vivo. Finally, we analyzed 2-bp insertions at the junctions of chromosomal rearrangements mediated by Cas9 programmed with various pairs of 14 additional sgRNAs and found that in all cases, except one for *HOXD12*, the most abundant 2-bp insertion is the same as the −5 and −4 positions of the noncomplementary strand upstream of the NGG PAM site (Supplementary Figs. S3 and S4). These data strongly suggest that Cas9 endonucleolytically cleaves at the −5 position of the noncomplementary strand upstream of the NGG PAM site in vivo, generating staggered ends with 2-bp overhangs.

In addition to 2-bp insertions, we also analyzed 3-bp insertions and found that, among the 27 observed insertion types, the most predominant 3-bp insertion type of “ACC” at the deletion junction is the same as the tri-nucleotide at the −6, −5, and −4 positions of the noncomplementary strand upstream of the NGG PAM site in vivo (Fig. 1k). This suggests that Cas9 also endonucleolytically cleaves at the −6 position of the noncomplementary strand upstream of the NGG PAM site in vivo, generating staggered ends with 3-bp overhangs.

To investigate the mechanisms of Cas9 cleavage in vitro, we cleaved a synthetic DNA fragment of *HOXA1* by the recombinant Cas9 proteins. We then analyzed diverse cleavage patterns of the noncomplementary strand by deep sequencing the library of the cleaved products (Supplementary Table S1). We observed that 85.21% reads are resulted from the RuvC cleavage at the −4 position of the noncomplementary strand upstream of the NGG PAM site (Fig. 1m; Supplementary Fig. S2f), confirming our previously observed cases of the predominant cleavage at the −4 positions in vivo^1^. By contrast, there are only 14.55% reads of blunted ends resulted from the cleavage at the −3 position (Fig. 1m; Supplementary Fig. S2f). We also observed that 6973 and 264 reads, corresponding to 0.23% and 0.009% of the total sequenced reads, respectively, are resulted from Cas9 endonucleolytic cleavage at the −5 and −6 positions of the noncomplementary strand (Supplementary Table S1). We also sequenced the library of the complementary strand, as controls, from the cleaved products and found that all of the sequenced reads are resulted from the precise HNH cleavage at the −3 position (Fig. 1m, n; Supplementary Fig. S2c–e). In addition, we observed remarkably similar cleavage patterns in vitro for the *β-globin* (Fig. 1n; Supplementary Fig. S2g), *HOTTIP* (Supplementary Fig. S2c, h), *RPA* (Supplementary Fig. S2d, i), and *CTCF* (Supplementary Fig. S2e, j) loci. To our knowledge, this is the first direct evidence for staggered Cas9 endonucleolytic cleavage at the −5 and −6 positions of the noncomplementary strand upstream the NGG PAM site. We propose a fill-in model of the diverse DSB ends from the Cas9 endonucleolytic cleavage for the predictable CRISPR di- or tri-nucleotide insertions (Fig. 1o).

It is puzzling that there is no substantive evidence of 2-bp insertions in recent studies of template-free CRISPR/Cas9 editing with single sgRNAs^2,3^. To this end, we sequenced 160 amplicon libraries of DSB repair outcomes from CRISPR/Cas9 editing with single sgRNAs for 22 sites and found that 16 sites have 2-bp insertions (Supplementary Figs. S5 and S6) and the other 6 sites do not have 2-bp insertions. Remarkably, among all of these 16 single-sgRNA-cleavage sites that have 2-bp insertions, the inserted dinucleotides all have strong bias toward the −5 and −4 positions on the noncomplementary strand (Supplementary Figs. S5 and S6). These data demonstrated that 2-bp insertions by CRISPR/Cas9 with single sgRNAs are also predictable. However, in almost all of these 16 cases the frequencies of 2-bp insertions by Cas9 with single sgRNAs are much lower than the corresponding 2-bp insertions at the junctions of DNA-fragment editing guided by dual sgRNAs (Supplementary Tables S2 and S3). These data strongly suggest that there exists frequent re-cutting after precise repair of Cas9 cleavage with single sgRNAs, because the two cohesive DSB ends from single cleavages are always complementary, while any two chromosomal rearranged DSB ends with distinct overhangs from dual cleavages are rarely complementary. Finally, the two DSB ends with 2-bp cohesive overhangs are much easier to be precisely re-ligated together than those with cohesive 1-bp overhangs. This explains why there are only reported 1-bp predictable insertions by Cas9 cleavage guided with single synthetic sgRNAs^2,3^.

In summary, we obtained strong evidence for biased 2-bp and 3-bp insertions resulting from Cas9 cleavage at the −5 and −6 positions, respectively, of the noncomplementary strand upstream of the NGG PAM site. Our data demonstrated that, in addition to the endonucleolytic cleavage at the −4 and −3 positions, Cas9 also endonucleolytically cleaves at the −5 and −6 positions of the noncomplementary strand upstream of the NGG PAM site and thus provide significant insights into the mechanisms of Cas9 cleavage. This finding lays the foundation for precise and predictable 2-bp or 3-bp insertional genome editing and points to a direction for future therapeutic corrections of disease alleles with 2-bp or 3-bp deletions.

## Supplementary information

Supplemtary Cas9 has no Exonuclease Activity Resulting in Staggered Cleavage with Overhangs and Predictable Di- and Tri-nucleotide CRISPR Insertions without Template Donor

## Data Availability

The deep sequencing data are available from the Sequence Read Archive (SRA) accession number PRJNA551796.

## Supplementary information

**Supplementary Information** accompanies the paper at (10.1038/s41421-019-0120-z).
