# Supplementary material for: Cas9 has no exonuclease activity resulting in staggered cleavage with overhangs and predictable di- and tri-nucleotide CRISPR insertions without template donor
Source: Cell Discov. 2019 Oct 1;5:53. doi: 10.1038/s41421-019-0120-z (PMC6796948; doi:10.1038/s41421-019-0120-z)
Supplement: Supplementary file 1 — Supplemtary Cas9 has no Exonuclease Activity Resulting in Staggered Cleavage with Overhangs and Predictable Di- and Tri-nucleotide CRISPR Insertions without Template Donor [file 41421_2019_120_MOESM1_ESM.pdf]

Supplementary Information

The file includes 6 Figures and 3 Tables as well as Materials and Methods.

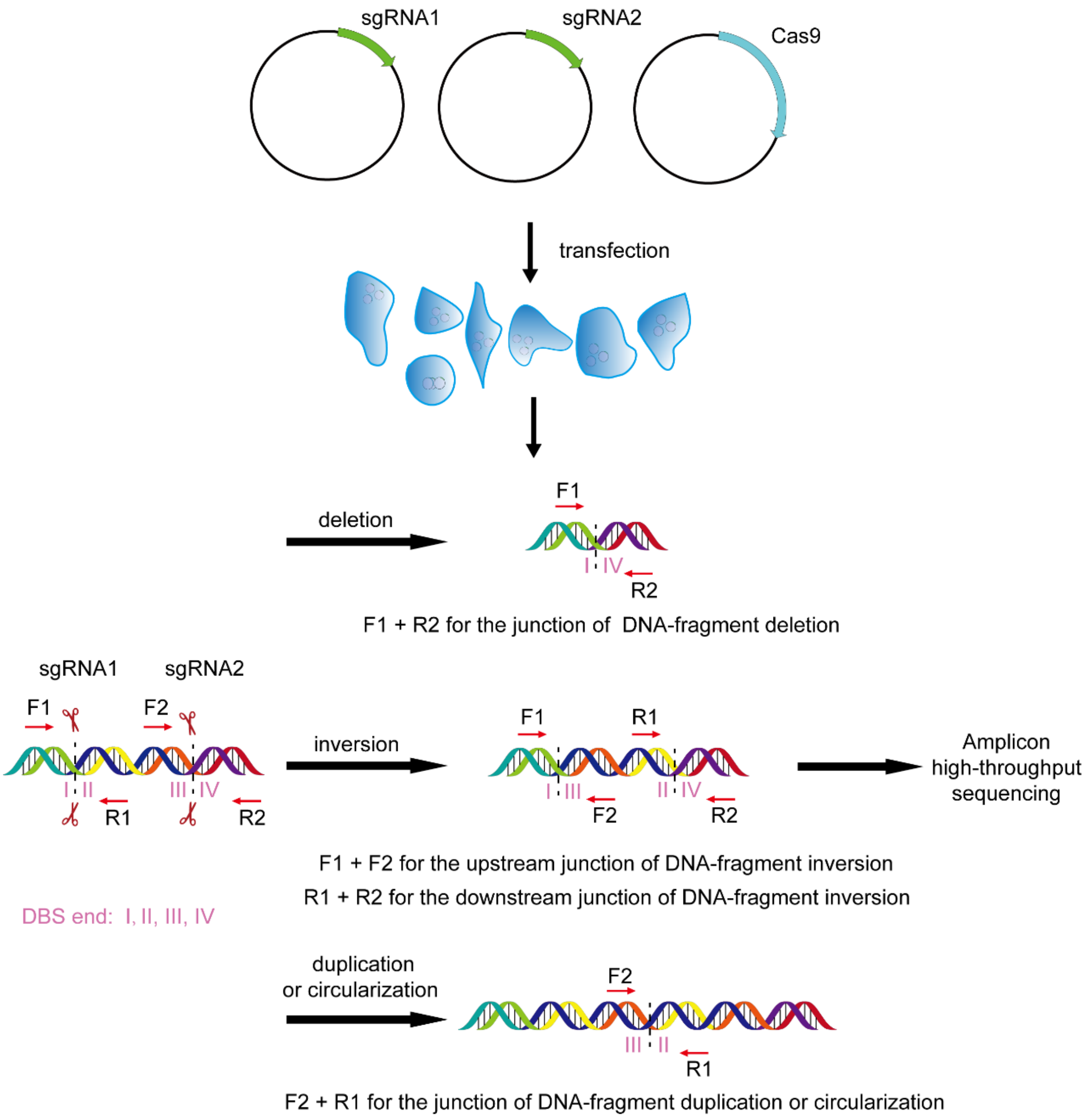

Figure S1. A pipeline for high-throughput measuring of repair outcomes of 2-bp and 3-bp insertions at the junctions of chromosome rearrangements with dual sgRNAs. After co-transfection of three plasmids of dual sgRNAs and Cas9, the junctions of chromosomal rearrangements including deletion, inversion, and duplication (circularization) are amplified by barcoded and indexed PCR primer pairs. The junctional amplicons are then sequenced *en masse* on an Illumina HiSeq X Ten platform. 2-bp and 3-bp insertions are analyzed after de-multiplexing.

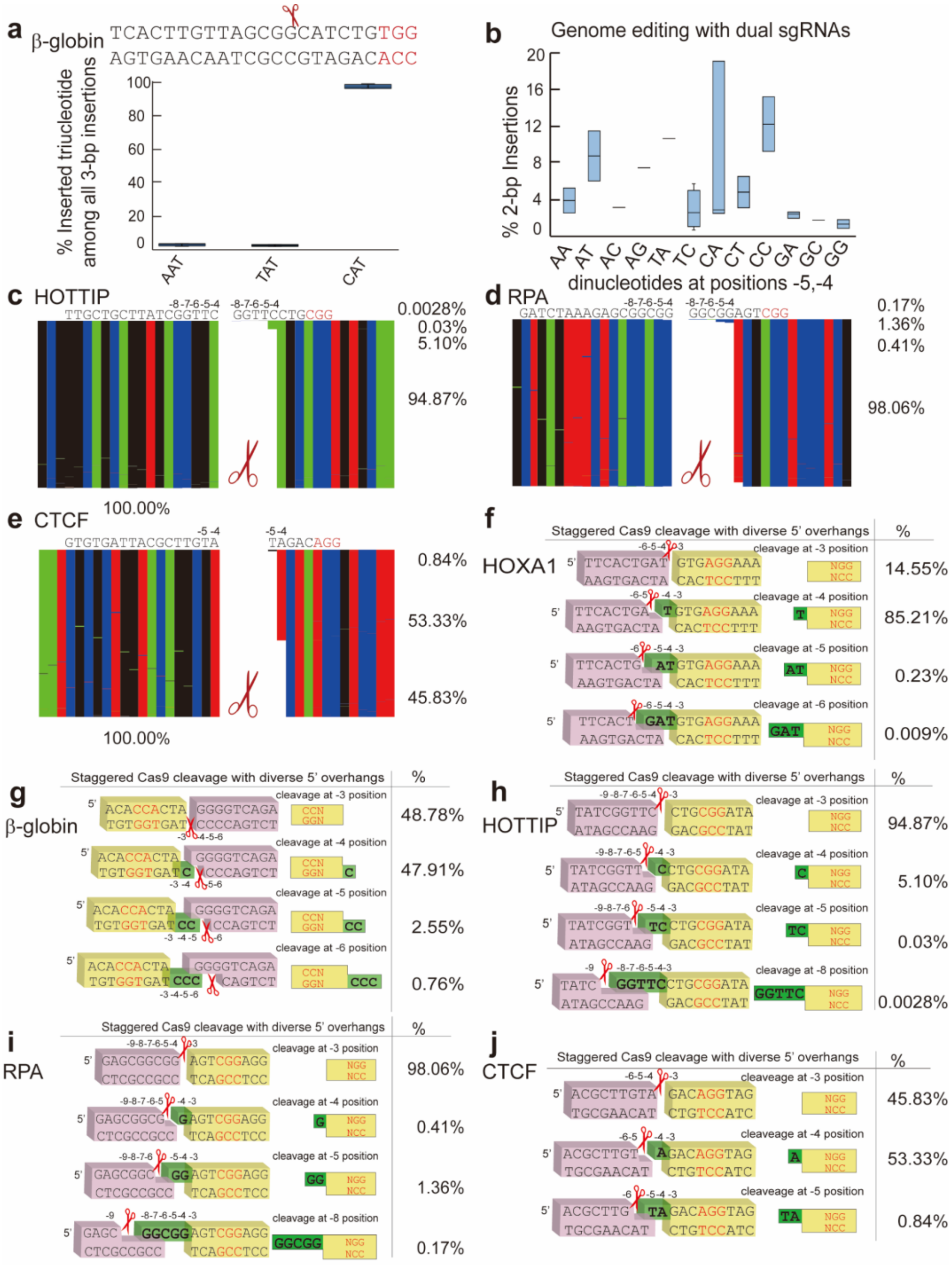

Figure S2. Di- or tri-nucleotide insertion patterns *in vivo* and Cas9 staggered cleavage profiles *in vitro*. a, 3-bp insertion frequencies of different trinucleotides in a *β-globin* targeting site. **b**, 2-bp insertion frequencies at diverse targeting sites with different dinucleotides at the -5 and -4 positions. The boxplot shows the upper quartile, the median, and the lower quartile. Whiskers show the 1.5 times of IQR (Interquartile Range). **c-e**, NGS raw sequencing reads showing staggered Cas9 *in-vitro* cleavage patterns of the targeting sites of *HOTTIP* (**c**), *RPA* (**d**), and *CTCF* (**e**). **f-j,** Illustration of staggered Cas9 *in-vitro* cleavage patterns of the targeting sites of *HOXA1* (**f**), *β-globin* (**g**), *HOTTIP* (**h**), *RPA* (**i**), and *CTCF* (**j**). The targeting sequences for all sites are shown with PAM highlighted in red.

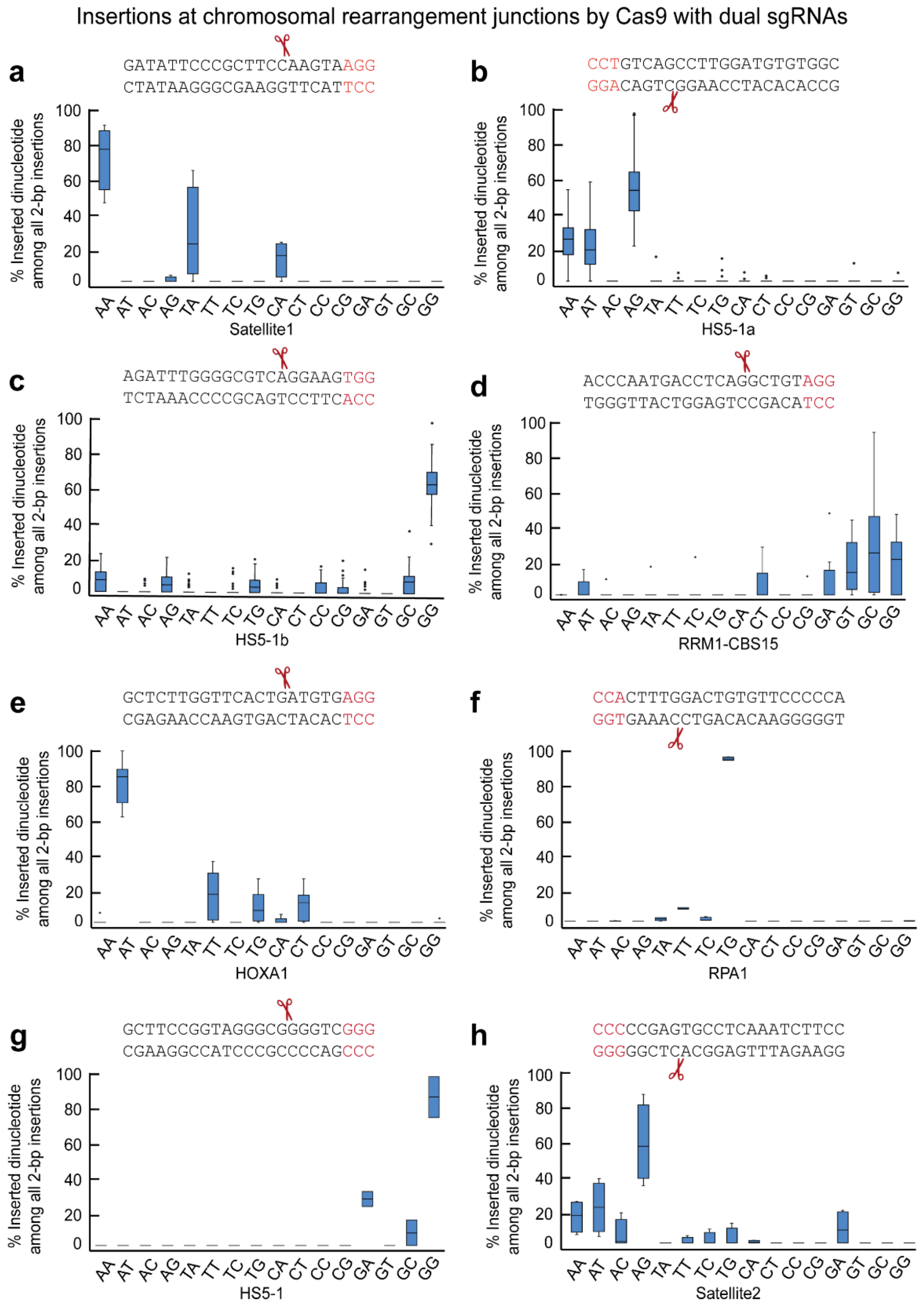

Figure S3. The 2-bp insertions at junctions of chromosomal rearrangements by Cas9 with dual sgRNAs have a strong bias toward dinucleotides at the -5 and -4 positions. a-h, Shown are boxplots of normalized 2-bp insertions frequencies for sgRNAs targeting the satellite1, a satellite region in human chromosome 11 (**a**), the *HS5-1a* (**b**) and *HS5-1b* (**c**) CTCF-binding sites, the ribonucleotide reductase large subunit 1 (*RRM1-CBS15*) gene (**d**), *HOXA1* (**e**), human replication protein A1 (*RPA1*) gene (**f**), the *HS5-1* enhancer (**g**), and the satellite2, a satellite region in the human chromosome 11 (**h**). The PAM sites are highlighted in red.

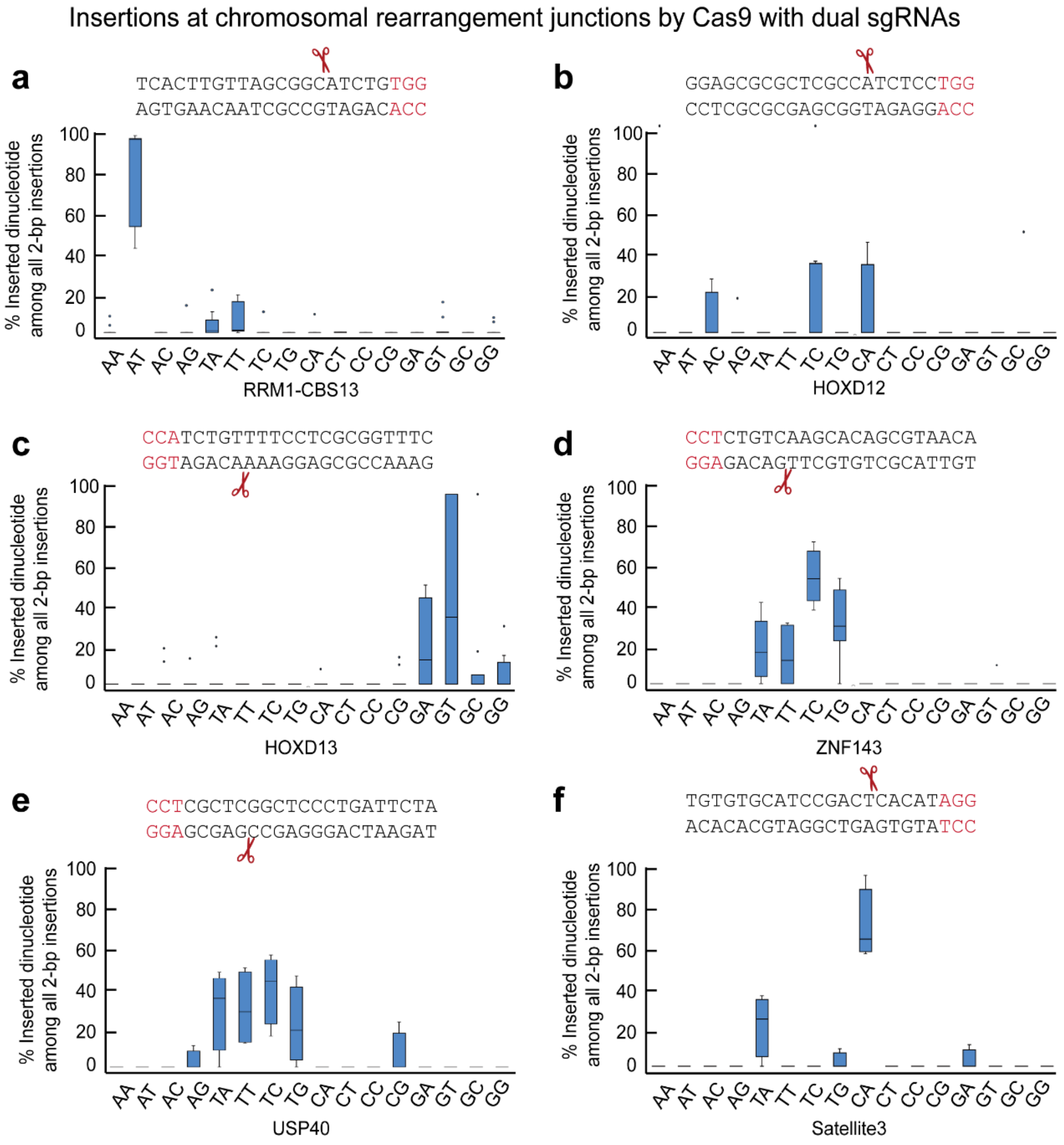

Figure S4. The 2-bp insertions at junctions of chromosomal rearrangements are biased toward -5 and -4 positions. **a-f**, Shown are boxplots of normalized 2-bp insertions frequencies for sgRNAs targeting the intron 14 of ribonucleotide reductase large subunit 1 (*RRM1-CBS13*) gene (**a**), *HOXD12* (**b**), *HOXD13* (**c**), a zinc-finger transcription factor *ZNF143* (**d**), ubiquitin specific peptidase 40 (*USP40*) gene (**e**), and the satellite3, a satellite region in the human chromosome 11 (**f**). The PAM sites are highlighted in red.

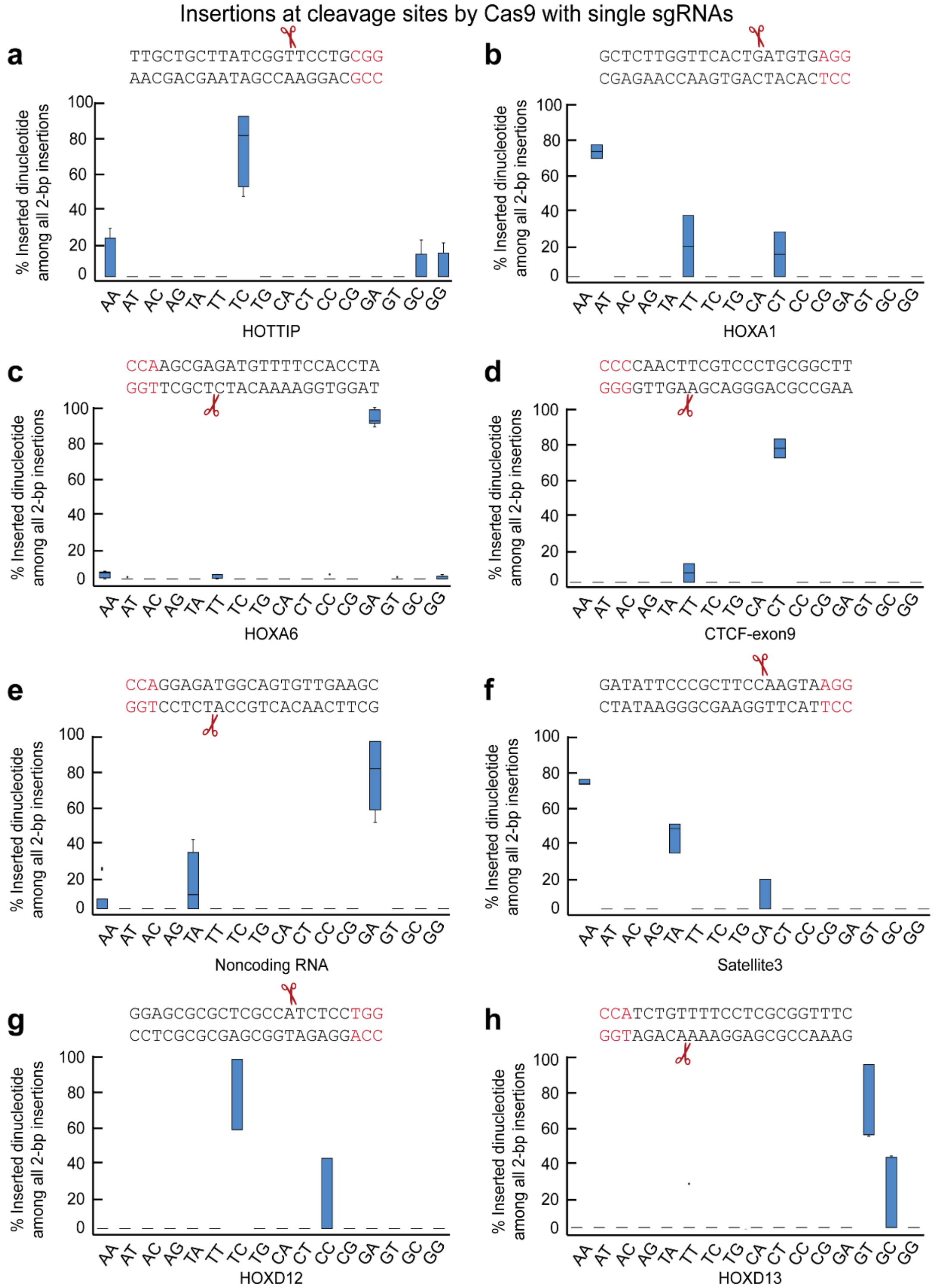

Figure S5. The 2-bp insertions of CRISPR/Cas9 editing with single sgRNAs are predictable. **a-h**, Shown are boxplots of normalized 2-bp insertions frequencies for sgRNAs targeting the human “*HOXA* transcript at the distal tip” antisense RNA (*HOTTIP*) (**a**), *HOXA1* (**b**), *HOXA6* (**c**), *CTCF-exon9* (**d**), a noncoding region near the *β-globin* cluster in the human chromosome 11 (**e**), the satellite 3, a satellite region in human chromosome (**f**), *HOXD12* (**g**), and *HOXD13* (**h**). The PAM sites are highlighted in red.

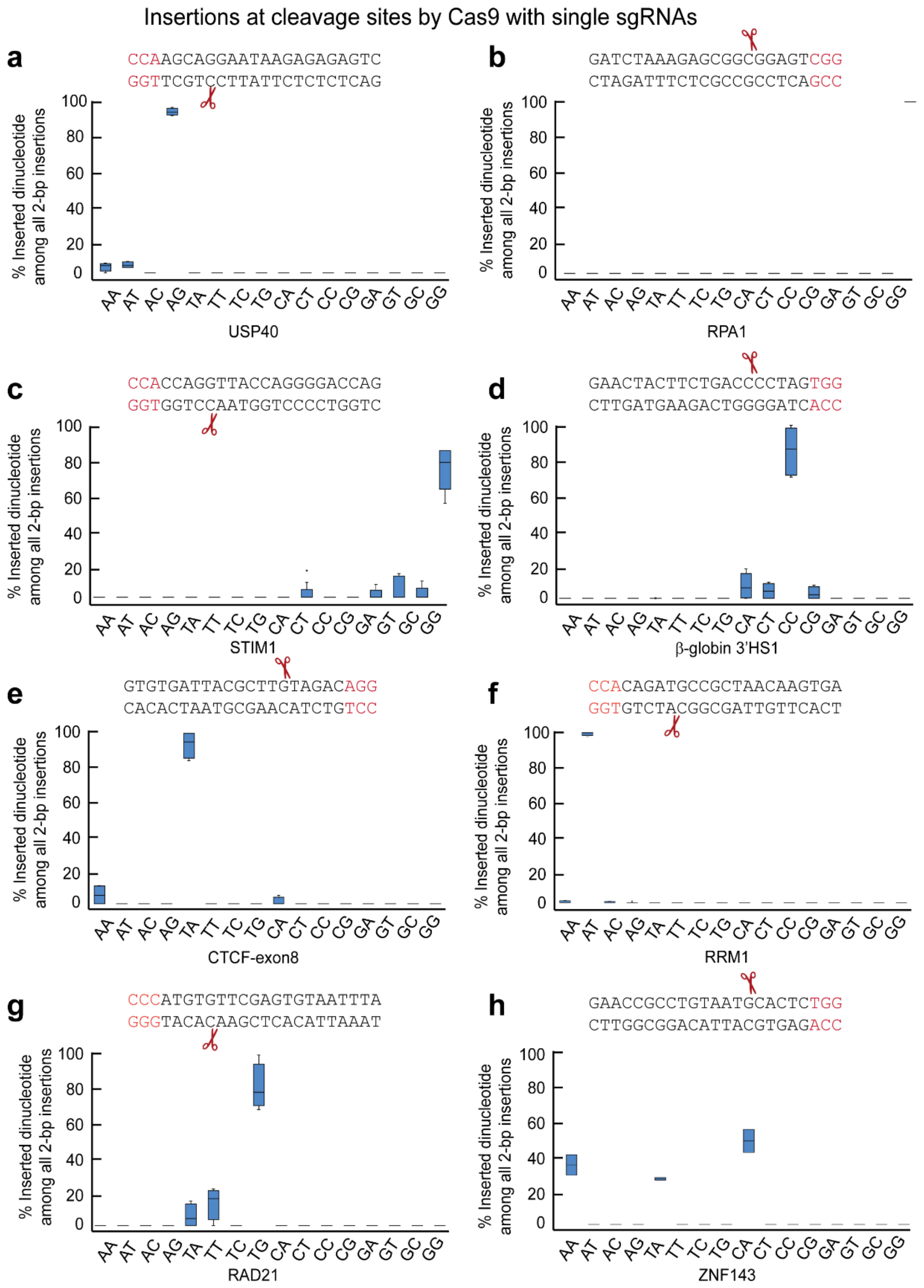

Figure S6. The 2-bp insertions of Cas9 editing with single sgRNAs are biased toward the dinucleotides at the -5 and -4 positions. **a-h**, Shown are boxplots of normalized 2-bp insertions frequencies for sgRNAs targeting *USP40* (**a**), *RPA1* (**b**), the intron of stromal interaction molecule 1 (*STIM1*) gene (**c**), *β-globin 3'HS1* (**d**), *CTCF-exon8* (**e**), the intron of ribonucleotide reductase large subunit 1 (*RRM1*) gene (**f**), *RAD21* (**g**), and a zinc-finger transcription factor *ZNF143* (**h**). The PAM sites are highlighted in red.

Table S1 Deep sequencing statistics of Cas9 *in-vitro* cleavage

sgRNA targeting site	Total Reads	-3 position	%	-4 position	%	-5 position	%	-6 position	%	-8 position	%
HOXA1	3006882	437645	14.555	2562000	85.205	6973	0.232	264	0.009	0	0.000
β-globin	711224	346905	48.776	340768	47.913	18150	2.552	5401	0.759	0	0.000
HOTTIP	2411913	2288108	94.867	122963	5.098	775	0.032	0	0.000	67	0.003
RPA	681527	668323	98.063	2812	0.413	9258	1.358	0	0.000	1134	0.166
CTCF	2574284	1179832	45.831	1372761	53.326	21691	0.843	0	0.000	0	0.000

Table S2 Deep sequencing statistics for chromosomal rearrangements by CRISPR/Cas9 editing with dual sgRNAs

sgRNA targeting site	Total Reads	Insertion Reads	% Insertion	2-bp Insertion Reads	% 2-bp Insertion	% 2-bp Insertion in all Insertions
CTCF-sgRNA1	1,811,187	1,431,381	79.03	178,771	9.87	12.49
CTCF-sgRNA2	4,139,895	460,647	11.13	68,267	1.65	14.82
RAD21-sgRNA1	4,048,984	412,463	10.19	38,602	0.95	9.36
RAD21-sgRNA2	5,756,106	1,275,209	22.15	146,291	2.54	11.47
STIM1-sgRNA1	53,496,696	32,348,190	60.47	920,860	1.72	2.85
STIM1-sgRNA2	70,675,853	15,164,564	21.46	1,529,814	2.16	10.09
Noncoding RNA	40,829,044	3,014,728	7.38	332,150	0.81	11.02
RRM1-CBS13	10,906,941	5,297,531	48.57	967,669	8.87	18.27
β-globin 3'HS1	25,606,555	10,723,485	41.88	2,786,513	10.88	25.99
HS5-1a	28,368,110	20,394,162	71.89	697,825	2.46	3.42
HOXA6	6,603,140	1,772,391	26.84	150,002	2.27	8.46
RRM1-CBS15	3,244,185	675,102	20.81	25,951	0.80	3.84
HS5-1b	24,493,939	6,479,042	26.45	234,005	0.96	3.61
HOXA1	2,247,936	1,156,085	51.43	107,843	4.80	9.33
RPA1	4,388,153	1,787,829	40.74	631,711	14.40	35.33
HS5-1SP	1,052,226	225,679	21.45	4,106	0.39	1.82
Satellite1	1,331,445	960,564	72.14	25,170	1.89	2.62
Satellite2	1,649,964	968,555	58.70	85,725	5.20	8.85
HOXD12	6,187,483	2,840,635	45.91	68,262	1.10	2.4
HOXD13	6,718,235	262,623	3.91	15,208	0.23	5.79
ZNF143	6,594,352	2,659,950	40.34	96,514	1.46	3.63
USP40	2,130,576	506,657	23.78	28,114	1.32	5.55
Satelite3	3,553,723	946,755	26.64	38,304	1.08	4.05
HOTTIP	7,682,781	1,772,726	23.07	312,571	4.07	17.63

Table S3 Deep sequencing statistics for CRISPR/Cas9 editing with single sgRNAs

sgRNA targeting site	Total Reads	Insertion Reads	% Insertion	2-bp Insertion Reads	% 2-bp Insertion	% 2-bp Insertion in all Insertions
Noncoding RNA	2,968,217	316,834	10.67	12,975	0.44	4.10
β-globin 3'HS1	4,013,764	901,292	22.46	496,183	12.36	55.05
RRM1	1,980,661	753,150	38.03	219,518	11.08	29.15
STIM1	4,374,696	321,301	7.34	10,631	0.24	3.31
CTCF-exon 8	487,113	190,506	39.11	5,669	1.16	2.98
CTCF-exon 9	1,186,571	54,313	4.58	3,129	0.26	5.76
Satellite1	2,157,339	323,124	14.98	3,751	0.17	1.16
HOXA1	2,079,092	965,112	46.42	2,359	0.11	0.24
HOXA6	1,305,843	213,867	16.38	48,440	3.71	22.65
HOTTIP	929,846	566,922	60.97	3,721	0.40	0.66
RAD21	558,134	192,342	34.46	7,266	1.30	3.78
RPA1	199,454	34,612	17.35	2,491	1.25	7.20
USP40	881,193	181,416	20.59	5,099	0.58	2.81
HOXD12	1,841,985	327,471	17.78	2,822	0.15	0.86
HOXD13	3,206,560	12,113	0.38	309	0.01	2.55
ZNF143	2,198,816	122,694	5.58	2,556	0.12	2.08

Materials and Methods

Cas9 cleavage *in vitro* and deep sequencing

The cleavage substrates were amplified by PCR with a specific pair of primers. Primers were designed to amplify the DNA fragment from the targeting genomic region, ensuring that after Cas9 cleavage, the two cleaved products could be separated easily by gel electrophoresis (*RPA1* Fw: 5'-CCAGC TTCAG CCGAC ATGAC-3', Rv: 5'-GTCAC CCCTG CTCCA AGATC-3'; *CTCF* Fw: 5'-GCATG AACCT GGGAA GTGGAG-3', Rv: 5'-CACCG GGATG ACACA ATTGAC-3'; *β-globin* Fw: 5'-TCGTA TCCCC TCTGA GCACTG-3', Rv: 5'-TCAGG TAAAC TGGAA ATTTA AGCC-3'; *HOXA* Fw: 5'-TGTGG CATGG AATTA GAACT GTG-3', Rv: 5'-GGATA CTTCC TGGGT CAAGT GC-3'; *HOTTIP* Fw: 5'- CAGCC TAGCT CAGAA TGGGTG-3', Rv: 5'-TCGCT CGCTC TATCT CAAAG TC-3').

SgRNA templates for *in vitro* transcription were amplified by a forward primer with T7 promoter sequence and a reverse primer matching scaffold sequences. SgRNAs were transcribed *in vitro* using the MEGAshortscript T7 kit (Life Technologies) and purified by the MEGAclear kit (Life Technologies). Cas9 proteins (0.75 μM) and in-vitro-transcribed sgRNAs (0.75 μM) were pre-incubated at 37°C for 20 min in the cleavage buffer (20 mM HEPES, 100 mM NaCl, 5 mM MgCl2, 0.1 mM EDTA, pH 6.5), then the targeting DNA (0.25 μM) was added and incubated at 37°C for 2 hours. The reactions were stopped by the addition of RNase A followed by proteinase K digestion. The cleaved DNA

products were run on 2% TBE-agarose gel and gel-purified by QIAGEN columns.

To sequencing each cleaved DNA product resulted from Cas9 cleavage, DNA libraries were constructed and deep sequenced on the Illumina HiSeq X Ten platform. Briefly, 20 ng of each Cas9 cleaved products were end-modification by Klenow Fragment (exo^-) and ligated with the NEBNext adaptor (NEB) followed by USER enzyme digestion. The adaptor-ligated DNA were purified by AMPure XP beads (Beckman) and amplified by PCR using the NEBNext Q5 DNA polymerase. The PCR conditions were as following: 60 seconds at 98°C for initial denaturation, followed by 15 cycles of 15 seconds at 98°C for denaturation and 60 seconds at 65°C for annealing and extension. The final extension step was 5 min at 65°C. The PCR products were purified by the High Pure PCR product purification kit (Roche) and quantified by a Qubit 3.0 Fluorometer (Life Technologies) for deep sequencing.

Cell culture. The human embryonic kidney 293 (HEK293T) cells were cultured in the Dulbecco's modified Eagle's medium (DMEM) supplemented with 10 % (v/v) FBS (fetal bovine serum) and 1% penicillin-streptomycin at 37 °C in a 5% (v/v) CO_2 incubator.

Construction of plasmids. The sequences of sgRNAs were designed according to the genomic sequences of the targeting genes or noncoding

regions, mostly on DNase I hypersensitive sites. For each targeting site, two 24-mer oligonucleotides with the first four bases as “5’ACCG” and “5’AAAC” respectively, and the following 20 nucleotides complementary to each other. After annealing, the double-stranded DNA ends with 5' overhangs of "ACCG” and “AAAC” were ligated into the linearized pGL3-U6 (Dr. Xingxu Huang from ShanghaiTech University) backbone which was purified after digestion with Bsa I. The Cas9-expressing plasmid pcDNA3.1-Cas9 was a gift from Dr. Jianzhong Xi from Peking University.

PAM configurations. We used two plasmids that express dual sgRNAs targeting specific sites flanking a DNA fragment with either NGG or CCN PAM sequences. Therefore, there are four combinations of paired PAM configurations (NGG-NGG, NGG-CCN, CCN-NGG, and CCN-CCN) in total. The 2-bp insertions at particular junctions of DNA-fragment editing are dependent on each of the four specific PAM configurations.

Mammalian cell transfection. HEK293T cells were defrosted from liquid nitrogen and plated on Petri dishes for passages when reaching 80-90% confluence. After plated in 12-well plates for 24 hours at an 80-90% confluence, the cells were co-transfected by Lipo3000 reagents with three plasmids, two of which express dual sgRNAs targeting two sites flanking a DNA fragment while the third one expresses the Cas9 endonuclease. After continuing culture for two

additional days, puromycin was added to a final concentration of 2 μg/ml and the cells were selected for four additional days.

Genomic DNA preparation. After collecting the cells by centrifuge at 6,000 rpm for 3 min and removing the supernatant, the cell pellet was re-suspended with the lysis buffer (25 mM NaOH with 0.2 mM disodium EDTA). Cells were then incubated at 98 °C for 40 min. Equal volume of the 40 mM Tris–HCl solution (pH = 5.0) was then added to neutralize the buffer. The genomic DNA was extracted by adding equal volume of isopropanol followed by spooling with glass fibers, washed twice in 70% ethanol, centrifuged, and re-suspended in TE. The genomic DNA can be stored at -20℃ or be used as templates to perform polymerase chain reaction (PCR) experiments.

Preparation of junctional amplicon libraries for deep sequencing. Four DSB (double-strand break) ends (I, II, III, and IV) were resulted from the two cleavage sites by Cas9 with dual sgRNAs (Supplementary Fig. S1). Chromosomal rearrangements including DNA-fragment deletion, inversion, and duplication could be generated after combinatorial ligations of these four DSB ends by cellular DNA repair machineries (Supplementary Fig. S1). For example, ligation of DSB ends I and IV results in DNA-fragment deletion. Ligations of DSB ends I and III as well as ends II and IV result in DNA-fragment inversion. Finally, ligation of DSB ends III and II results in DNA-fragment duplication or

circularization (Supplementary Fig. S1). PCR amplification of these ligated junctions could be used to identify appropriate DNA repair outcomes of chromosomal rearrangements of deletion, inversion, and duplication. A pair of PCR primers were used to obtain the amplicon libraries for each ligated junction.

High throughput deep sequencing. PCR was performed using a pair of primers with Illumina P5 and P7 adapters and barcodes or indexes by the high-fidelity DNA polymerase to amplify the junctions of chromosomal rearrangements. The PCR conditions are as following: initial denaturation at 94 °C for 4 min, 35 cycles of denaturation at 94 °C for 30 s, annealing at 60 °C for 30 s and extension at 72 °C for 50 s, and followed by the final extension at 72 °C for 3 min. The structure of the constructed library is as following: 5' universal sequence - P5 adapter - barcode - DNA insert - P7 adapter - index - 3' universal sequence. Therefore, the structure of the forward primer should be the 5' universal sequence - P5 adapter - barcode - specific primer, and the reverse primer should be the 5' universal sequence - index - P7 adapter - specific primer. The P5 and P7 universal sequences are used to anchor amplicons to the flow cell. The index, either 6-bp or 8-bp, is used to distinguish samples from different sources. The barcode, usually 4-bp in length, is used to distinguish different replicates. After purification of PCR amplicons and determination of their concentrations, the libraries are pooled and denatured for sequencing on the HiSeq X Ten platform with 150-bp reads. In order to cover

the junction site, one primer should be close to the junction site, but with the distance more than 50 bp. To obtain appropriate amplicon libraries of 300 - 500 bp, the other primer was designed to be further away from the junction with the distance of 250 - 450 bp. The sequencing raw data were then de-multiplexed according to the barcodes and indexes and the adapter sequences were trimmed.

Analyzing junctions of editing. To analyze editing outcomes and find indels, the de-multiplexed sequencing data were first sorted via a customized program. By sorting, the frequencies of each type of sequencing reads were obtained and the sequencing reads with frequencies below 0.02 % were excluded. Then the sequences were arranged according to their frequencies and their corresponding number of reads were also displayed. In order to find indels (deletions and insertions), the sorted data were compared with their corresponding reference sequence via an Indel-calling program. The different bases were showed with their corresponding frequencies and number of reads. For our analyzation, the 2-bp insertions located exactly at junctions were counted. The nucleotide insertions and their frequencies were recorded.

The software program. The program used for sorting the raw reads is written in C++. Not only can it sort the reads of the FASTQ files into different sequence types, but it can also sort the reads in the TXT format. Information including

sequence types, reads numbers and frequencies can be generated by running the program. A patent based on this program has been filed. Sorting the sequences from the highest to the lowest frequency depends on the quick sort algorithm. This program excludes reads that are less than a frequency of a settable threshold.

The Indel-calling program is written in Python, which can compare the sorted data with the reference sequences, and thus find the differences. The single base differences that do not overlap with the junction site is assumed to be SNPs or sequencing errors. If there exist gaps in sequencing data, this program defines reads as deletions. If the gaps are in references, then the reads are defined as insertions. If the sequence is the same as the reference, then the reads are precise ligations. This program also displays the number of reads and the corresponding frequency of each reads type. The positions where differences of bases occur are also recorded.

2-bp insertion analysis. The data processed by the Indel-calling program will display the indel types as well as the inserted or deleted nucleotides, the corresponding frequencies, and the total number of reads. We focused on the cases of 2-bp insertions. Only the 2-bp insertions at the ligation junctions are considered. The final results are shown in box plots.

Manual inspection of disambiguation. Because the program is designed to assure the maximum matches of the downstream sequences between a read and the ligation reference, it could assign the incorrect location for the inserted bases if they match the flanking sequences. In these cases, we manually inspect the reads and assign the insertion location to the ligation junctions.

Probability logo. DNA motif for 2-bp insertions was obtained via the k-mer probability logo (*k*pLogo) with default parameters except that the k-mer length equals to 1. Enrichment and depletion of nucleotides at each position is shown above and below the position coordinates, respectively. Base positions with Bonferroni-corrected P values (< 0.01) are highlighted.

Statistical analysis. In our study, 731 libraries in total were analyzed in which 571 libraries were analyzed for dual sgRNAs (558 libraries were from SRA database: SRP144399) and 160 libraries (SRA database: SRP144399) were analyzed for single sgRNAs. Each junction was sequenced with at least two biological replicates. The 2-bp insertion frequencies were normalized. The data were shown in boxplots with the upper quartile, the median, and the lower quartile values. Lines out of the box called whiskers show the 1.5 times of IQR (Interquartile Range). Outliers are plotted as individual points. The significance of their differences was evaluated by two-sided Student's t-test. *$p < 0.05$, **$p < 0.01$ and ***$p < 0.001$.

The deep sequencing data are available from the Sequence Read Archive (SRA) accession number PRJNA551796.

The programs in this study are available in GitHub https://github.com/MackZhang/Indel.

The patent based on this work has been filed. Patent application number is CN201710802423.
